# Divergent respiratory modes drive differences in heat tolerance and habitat use among tropical intertidal crabs

**DOI:** 10.1242/jeb.251854

**Published:** 2026-04-22

**Authors:** Pedro Julião Jimenez, Lyle Dennis Vorsatz, Stefano Cannicci

**Affiliations:** ^1^ Swire Institute of Marine Sciences, The University of Hong Kong, Pokfulam Road, Hong Kong, Hong Kong SAR, China; ^2^ School of Energy and Environment, City University of Hong Kong, Hong Kong SAR, China; ^3^ State Key Laboratory of Marine Pollution, City University of Hong Kong, Hong Kong SAR, China; ^4^ Department of Biological Science, University of Cape Town, Cape Town 7701, South Africa; ^5^ South African Institute for Aquatic Biodiversity (SAIAB), Makhanda, South Africa; ^6^ Department of Biology, University of Florence, I-50019, Sesto Fiorentino, Italy

**Keywords:** Thermal physiology, Oxygen limitation, Metabolism, Physiological plasticity, Terrestrial adaptation, Vertical distribution, Thermal limits

## Abstract

Physiological adaptations for amphibious living underpin successful sea-to-land transitions and shape thermal tolerance. Intertidal brachyurans crabs independently evolved contrasting respiratory strategies: air-breathing lungs in fiddler crabs (Gelasiminae) and water-dependent gill respiration in sentinel crabs (Macrophthalmidae). How these strategies influence thermal performance, vulnerability to climate change, and habitat use remains poorly understood. We examined whether air- versus water-breathing strategies affect the oxygen delivery and upper thermal limits in closely related intertidal species, *Macrophthalmus tomentosus* (Macrophthalmidae) and *Tubuca arcuata* (Ocypodidae). We measured cardiac performance, oxygen consumption (*Ṁ*_O_2__) and haemolymph oxygen partial pressure (*P*_O_2__) across 25–40°C in both air and water. The air-breathing *T. arcuata* exhibited higher upper lethal temperatures (mean±s.e.m.: 42.2±0.4°C versus 41.65±0.6°C) and maintained exponential increases in *Ṁ*_O_2__ when breathing air, despite inhabiting a cooler microhabitat. Additionally, *T. arcuata* sustained higher arterial and venous *P*_O_2__ during aerial heating, indicating efficient oxygen delivery near their thermal limits. In contrast, *M. tomentosus* was severely oxygen-limited during emersion, with 90% impaired recovery, and exhibited oxygen deficits under present-day warm habitat conditions, implying that extant populations operate near their physiological thresholds with minimal thermal safety margins. Both species showed constrained performance when submerged at high temperatures, indicating universal oxygen limitation in water. Our findings show that the evolution of air breathing improves oxygen delivery across environmental stressors, thereby enhancing aerobic scope and thermal resilience. Our approach provides a mechanistic explanation for both current habitat partitioning and differences in climate vulnerability among tropical intertidal crabs.

## Teaser

**Summary:** The sea–land transition exposed crabs to novel physiological stressors. Air breathing improved aerobic scope and thermal resilience in intertidal crabs facing heat, hypoxia and desiccation on land.

## INTRODUCTION

Metazoans have repeatedly transitioned from sea to land, often driven by factors such as oxygen limitations in aquatic environments (Giomi et al., 2014; Greenaway, 1999; Little, 1990). Terrestrial habitats offer greater oxygen availability, reducing the metabolic costs of respiration and increasing potential aerobic scope (the difference between minimum and maximum oxygen consumption rates), which in turn impacts an organism's energy budget (Giomi et al., 2014). Large aerobic scopes allow organisms to sustain higher activity levels and tolerate greater environmental stresses, such as temperature fluctuations, by improving oxygen uptake and diffusion (Bennett, 1978; Pörtner, 2010). The incentive of a larger aerobic scope was likely one of the drivers for the evolution of air-breathing adaptations in aquatic organisms (Giomi et al., 2014). This has led to multiple lineages independently converging on this breathing mode, resulting in varying degrees of dependence on aquatic environments (Burggren and McMahon, 1988; Greenaway, 1999).

However, intertidal and terrestrial habitats are subjected to larger variations in temperatures compared with fully aquatic environments. Such variations strongly influence the capacity for oxygen delivery and metabolic rates in all ectotherms (Amarasekare and Savage, 2012; Clarke, 2017; Pörtner, 2001). Each species has a thermal range within which aerobic scope is maintained (Angilletta, 2009; Pörtner, 2001; Pörtner et al., 2017). Beyond this range, increased temperatures raise oxygen demand, eventually leading to cellular hypoxia, a shift to anaerobic metabolism and loss of performance (Huey and Stevenson, 1979; Pörtner, 2010; Schulte, 2015). This concept underpins the oxygen- and capacity-limited thermal tolerance (OCLTT) framework, in which critical thermal limits are set by the capacity of the circulatory and respiratory systems to meet tissue oxygen demand, and failure of oxygen supply at thermal extremes can ultimately lead to mortality. However, OCLTT primarily focuses on individual thermal performance curves and does not directly address how interspecific variation in respiratory mode drives ecological niche partitioning and microhabitat segregation within thermally heterogeneous environments (Portner et al., 2017). This knowledge gap is further compounded by the fact that oxygen delivery capacity is constrained not only by organism physiology but also by medium properties: temperature-dependent changes in water viscosity alter oxygen diffusion rates and influence oxygen uptake efficiency (Verberk et al., 2022). Additionally, organismal traits such as body size and cell surface-area-to-volume ratios modulate the severity of oxygen limitation, potentially explaining differential thermal vulnerability among species of similar respiratory mode (Verberk et al., 2022). Whether respiratory mode (air breathing versus water breathing) maintains adequate oxygen delivery across media with contrasting properties and oxygen availability remains unexplored in sympatric tropical species, yet this interaction likely underlies niche partitioning and thermal vulnerability. Thus, examining whether physiological differences in oxygen delivery capacity mechanistically explain thermal performance becomes necessary to better understand the drivers for intertidal species distribution and evaluate the thermal vulnerability of bimodal-breathing organisms.

Different lineages within the Brachyura have evolved varying degrees of independence from fully aquatic respiration (Burggren and McMahon, 1988; Greenaway, 1988). Semi-terrestrial species exhibit a range of morphological and physiological adaptations that facilitate aerial respiration to enable survival inland (Burggren, 1992; Burggren and McMahon, 1988; Cannicci et al., 2008; Farrelly and Greenaway, 1993; Fratini et al., 2005; Henry, 1994; Morris, 2002). Brachyuran crabs play key ecological roles of sediment bioturbators and ecosystem engineers in soft-bottom intertidal habitats (Kristensen, 2008; Kristensen et al., 2012; Penha-Lopes et al., 2009). Among the families adapted to the intertidal environment, the Macrophthalmidae and the Ocypodidae have followed distinct evolutionary pathways towards terrestrialization (Chen et al., 2019; Schubart et al., 2006). Ocypodids have evolved branchiostegal lungs for direct aerial oxygen uptake (Greenaway and Farrelly, 1984; Paoli et al., 2015), whereas macrophthalmids rely on water ventilation in their gill chambers (Hawkins and Jones, 1982; Hawkins et al., 1982). As a result, macrophthalmids are constrained to low-intertidal, soft-bottom habitats (Nye, 1974; Schuwerack et al., 2006), whereas ocypodids occupy high-intertidal zones on sandy beaches, rocky shores and mangrove forests (Crane, 1975; Kwok and Tang, 2006; Takeda and Murai, 2003). Both low- and high-intertidal habitats are thermally stressful, and these crabs often experience temperatures near or beyond their thermal limits (Darnell et al., 2015; Jimenez et al., 2022). The evolutionary divergence, distinct habitats and contrasting respiratory strategies of these two families make them ideal models for exploring how primary breathing mode influences niche segregation, thermal tolerance and climate vulnerability of intertidal ectotherms.

In this study, we investigated how contrasting primary breathing modes affect aerobic metabolism and cardiac performance during acute temperature increases in both air and water. We expand on the OCLTT framework by examining whether oxygen delivery capacity, determined by respiratory mode, predicts thermal performance and microhabitat occupation in closely related intertidal crabs. Specifically, we compared the primarily air-breathing *Tubuca arcuata* (Ocypodidae) with the water-breathing *Macrophthalmus tomentosus* (Macrophthalmidae) to test mechanistic hypotheses about thermal tolerance and respiratory strategy. We hypothesized that air-breathing adaptations confer on *T. arcuata* a larger aerobic scope across thermal gradients than the water-breathing *M. tomentosus*, which is more constrained by oxygen availability at elevated temperatures. We also postulate that the expanded aerobic budget during aerial respiration in the air-breathing crab enables sustained oxygen delivery at elevated temperatures. Such enhanced oxygen delivery should grant *T. arcuata* both higher thermal tolerance limits and improved post-thermal-stress recovery, ultimately allowing this species to maintain aerobic performance during prolonged emersion in drier microhabitats. By combining respirometry, cardiac performance measurements and thermal tolerance assays, we aimed to elucidate the mechanistic basis by which respiratory mode influences thermal vulnerability and habitat segregation in intertidal ectotherms.

**Table:** 

**List of symbols and abbreviations**	
ABT	Arrhenius breakpoint temperature
CV	cross validation
CW	carapace width
LT	upper lethal temperature
MAE	mean absolute error
*Ṁ*_O_2__	oxygen consumption rate
OLS	ordinary least squares
OPT	optimum performance temperature
*P*a_O_2__	arterial blood partial pressure of oxygen
*P*_O_2__	blood partial pressure of oxygen
*P*v_O_2__	venous blood partial pressure of oxygen
RLM	robust MM-estimator
RMSE	root mean squared error
SESOI	smallest effect size of interest

## MATERIALS AND METHODS

### Sampling sites and model organisms

Specimens were collected from mudflat and mangrove habitats within Tung Chung wetlands (22°16′55″N, 113°55′40″E), Hong Kong Special Administration Region (SAR), China. *Macrophthalmus tomentosus* Eydoux & Souleyet 1842 and *Tubuca arcuata* (De Haan 1835) were selected as model species owing to their abundance, ecological relevance and distinct habitat preferences. *Macrophthalmus tomentosus* is found in low intertidal mudflats (Kostina et al., 2016; Sasekumar, 1974), whereas *T. arcuata* inhabits muddy substrates along tidal creeks and within mangroves, ranging from upper intertidal to supratidal zones (Kostina et al., 2016; Kwok and Tang, 2006; Lin et al., 2002). Both species are euryhaline, occupying estuarine environments characterized by fluctuating salinity owing to tidal exchange and freshwater input (Lin et al., 2002; Sasekumar, 1974).

### Sampling and acclimation

Sampling and experiments were conducted during the summer months (July to October). Only adults in the intermoult stage were collected; ovigerous females were excluded from the experiments owing to potential physiological effects associated with brooding behaviour (Baeza and Fernández, 2002; Fernández et al., 2000). Specimens were transported to the laboratories at the School of Biological Sciences, The University of Hong Kong.

Each species was maintained in separate tanks (41×56×78 cm, height×width×length) containing a ∼5 cm layer of aerated seawater (∼30 PSU) to prevent osmotic imbalances and hypoxia in *M. tomentosus* (Hawkins and Jones, 1982; Henry, 1991; Thurman, 1998). Tanks were tilted (∼15 deg) to allow the crabs access to both aquatic and aerial environments. No more than 20 individuals of each species were housed per tank, and water was changed weekly. We provided refuge and climbing substrate using 20 PVC pipe joints (32 mm diameter) per tank, mimicking the burrow structure found in their natural mangrove habitats.

Laboratory conditions were maintained at a 12 h:12 h light:dark photoperiod and a temperature of ∼25°C. To minimize lab-induced acclimation effects, crabs were kept in the maintenance tanks for no longer than 2 weeks prior to experimentation. Crabs were fed a diet of crushed Tropical Bionautic Flakes and Tropical Crusta Sticks. During the acclimation period, we monitored the boxes for signs of stress in the crabs. The absence of limb autotomy, mouth foaming or mortality, and the rare cases of aggressive behaviour among crabs indicated adequate maintenance conditions for both species. All animals underwent a 24-h fasting period before experiments to standardize metabolic conditions.

### Characterisation of habitat temperature

**Fig. 1.**
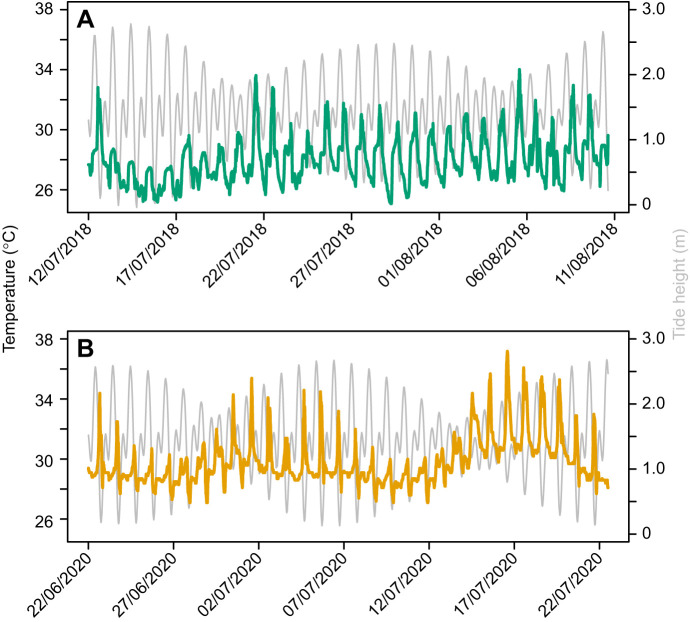
**Sediment surface temperatures experienced by the study species in their respective habitats (averaged iButton temperatures).** (A) *Tubuca arcuata* and (B) *Macrophthalmus tomentosus*. Green and orange lines represent the temperature experienced, colour-coded by species; grey lines represent the tide height.

To assess the local sediment surface temperatures (°C) experienced by the model species, Maxim Integrated iButton thermologgers were deployed at the collection sites during summer for approximately 1 month (June–July; deployment period in Fig. 1). The thermologgers were waterproofed using either Plasti Dip or 3M Scotchcast resin. Devices were placed in locations where crabs were most active, engaging in feeding, courtship, and burrow construction during low tides. Five thermologgers were deployed in the muddy low intertidal zone to characterize the habitat of *M. tomentosus*, and eight were placed within the mangrove forest area where *T. arcuata* are found.

### Determination of cardiac function and thermal limits

Experiments were conducted to determine cardiac performance in adult males of each model species (*n*=10 for each species, *N*=20 specimens). Specimens were immobilized on plastic grids using cable ties: one for each set of walking legs (left and right) and one for each cheliped. No appendage autotomy was observed after the crabs' legs were tied down. The specimens were then individually placed in 250 ml opaque blacked-out plastic containers with a layer of artificial seawater (Instant Ocean, 30 PSU) to a level reaching the ventral surface of the crabs, reducing the risk of water loss and its physiological effects (Burggren, 1992; Burggren et al., 1990).

Cardiac function was measured following Jimenez et al. (2022, 2025). Briefly, an infrared sensor (Vishay Semiconductors, CNY70) was attached to the carapace above the cardiac region using Blu Tack and cyanoacrylate glue. Heartbeat signals were recorded, filtered (Burnett et al., 2013) and transmitted to an oscilloscope (PicoScope 2204) connected to a computer, with data acquisition via PicoScope 7 software.

Crabs in individual containers were immersed in a temperature-controlled circulating water bath (Grant Optima TXF200, Cambridge, UK) and allowed to acclimate to immobilization and the initial experimental temperature (25°C) for 30 min following handling. Containers had the cap drilled to insert the infrared probe, and the hole was sealed with Blu Tack adhesive. The cap was always placed outside the water and was not tightly screwed, maintaining oxygenation inside the container. Crabs were then subjected to a thermal ramp, starting at 25°C and increasing by 1°C every 20 min until cardiac activity ceased. Heartbeats were recorded continuously throughout the thermal ramp. The initial temperature reflected laboratory maintenance conditions, and the rate was based on the 95th percentile of natural temperature variation in vegetated habitats in Tung Chung (Jimenez et al., 2022), ensuring ecological relevance.

To correct for potential differences between water bath and crab body temperatures, a protocol based on Jimenez et al. (2022) and Levinton et al. (2020) was employed. Ten adult males of each species (*N*=20), of similar size to those used in the heart function trials, were subjected to the same thermal ramp as described previously. A k-type thermocouple was inserted into the branchial chamber of the crabs and secured with Blu Tack and cyanoacrylate glue. The thermocouples were connected to a multichannel thermometer (Lutron TM-947SD) to record body temperature (±0.1°C) every minute, and the water bath temperature was logged concurrently. Body temperature was plotted against water temperature, and linear regressions were fitted for each species. These regressions were used to correct body temperature measurements in the cardiac function experiment.

### Determination of routine metabolic rate

To assess the routine metabolic rate of the model species in response to increasing temperature in both in air and water, oxygen consumption rate (*Ṁ*_O_2__) experiments were conducted on crabs subjected to a thermal ramp. Adult males from each species (*n*=10 per species for both aerial and aquatic respiration; total *N*=40) were blotted dry and cleaned with cotton swabs using distilled water followed by absolute ethanol prior to experimentation. Crabs were then dried to ensure no ethanol residuals remained on their carapace. Each crab was then placed in an individual, ethanol-sterilized, opaque acrylic chamber (380 ml), fitted with an oxygen spot sensor (Loligo Systems, Viborg, Denmark) affixed to the inner wall.

The chambers were submerged in a programmable water bath (Grant Optima TXF200). Prior to the start of each trial, crabs were allowed to recover from handling and acclimate to the initial experimental temperature (25°C) for 30 min. The thermal ramp was then initiated, increasing the temperature from 25°C to 40°C at a rate of 1°C every 20 min. *Ṁ*_O_2__ was measured at 25, 28, 31, 34, 37 and 40°C. These temperatures correspond to the average summer daily (28.9°C), the average daily maximum (31.6°C) and the averaged absolute maximum (34.4°C) temperatures recorded in Hong Kong from 2009 to 2018 (Hong Kong Observatory, http://www.hko.gov.hk). The 40°C measurement was included based on projected future temperatures increases (Calvin et al., 2023) and elevated heart rates observed in both species during preliminary cardiac performance experiments, which indicate heightened oxygen demand.

At each experimental temperature, the chambers were sealed, and two oxygen saturation measurements were taken for each chamber, one 5 min after sealing and another after 15 min, using a Witrox oxygen meter (Loligo Systems) connected via fibre optic cable to an oxygen spot. Data were logged using Witrox View software. Prior to each experiment, the Witrox oxygen meter was calibrated using oxygen-saturated water (100% saturation) by pumping air, and water depleted of oxygen (0% saturation) by adding sodium sulphite. *Ṁ*_O_2__ (expressed as µg O_2_ g^−1^ h^−1^) was calculated based on the change in oxygen saturation between measurements. To monitor chamber temperature, a probe was placed in an empty sealed chamber subjected to the same thermal ramp, and these readings were used in subsequent *Ṁ*_O_2__ calculations.

Blank chambers (*n*=5 for each medium, air and water) were used to control for background oxygen consumption in each trial. The absolute values of oxygen saturation changes recorded in the controls were subtracted from experimental values. Crab volume was measured by water displacement in a graduated cylinder and subtracted from the total chamber volume upon completion of experiments.

Following each trial, the survival of crabs was assessed by placing individuals in oxygen-saturated water for 5 min and subsequently testing their motor responses. Crabs were considered alive if they responded to tactile stimulation of the appendages and eyestalks.

During the aerial respiration experiments, oxygen saturation never dropped below 80% and relative humidity was maintained above 70%. For the aquatic respiration trials, aerated, UV-sterilized artificial seawater (30 PSU) was circulated through the chambers from a reservoir. The reservoir and chamber temperatures were matched and controlled using heating coils and a thermostat. Water oxygen saturation was maintained above 60% throughout aquatic *Ṁ*_O_2__ measurements to prevent hypoxia (Burke, 1979).

### Measurement of partial pressure of oxygen in haemolymph

To assess oxygenation in the haemolymph, individuals from both species were rinsed in seawater and blotted dry prior to experimentation. Crabs were immobilized as described previously, and the carapace was carefully drilled above the cardiac region to expose the pericardial sinus. No mortality or signs of stress were observed after drilling.

Each crab was placed individually into a 250 ml opaque blacked out plastic container. For aerial partial pressure of oxygen (*P*_O_2__) measurements, a thin layer of seawater (30 PSU) was added to the container, as described for cardiac function measurements. For aquatic *P*_O_2__ measurements, containers were filled with seawater (30 PSU) and aerated to prevent hypoxia. All containers were placed in a temperature-controlled water bath (same model as in previous experiments).

Crabs were allowed to recover and acclimatize for 30 min, then subjected to the same thermal ramp protocol used in the routine metabolic rate experiment. Once the desired experimental temperatures were reached, crabs were maintained at that temperature for 10 min prior to measuring. Arterial haemolymph was sampled from the pericardial sinus using modified Pasteur pipettes, and venous haemolymph was sampled from the arthrodial membrane at the base of the fourth walking leg.

The *P*_O_2__ in arterial and venous haemolymph was measured using an oxygen sensor connected to a Microx TX3 oxygen meter (PreSens Precision Sensing, Regensburg, Germany) with Microx integrated signal processing software. Prior to experimentation, the Microx TX3 oxygen meter was calibrated using water saturated with oxygen (100% saturation) by pumping air, and oxygen-depleted water (0% saturation) by addition of sodium sulphite. *P*_O_2__ (expressed as mmHg) was measured in both air and water at four temperatures: 28, 31, 34 and 40°C (eight individuals per species, per medium, per temperature; *N*=128).

Atmospheric pressure and room temperature were recorded at the time of measurements. Local atmospheric pressure data were obtained from the Hong Kong Observatory (www.hko.gov.hk), and room temperature was recorded using the Microx TX3 temperature probe. Haemolymph *P*_O_2__ was calculated based on these parameters.

All crabs used in the experiments were weighed with a precision balance (±0.001 g) and carapace width (CW) was measured using a dial calliper (±0.1 mm). Size and mass data are summarized in Table 1.

**Table 1.** Summary of the carapace width (CW) and mass of the crabs used in the experiments

Experiment	*Tubuca arcuata*		*Macrophthalmus tomentosus*	
	CW (mm)	Mass (g)	CW (mm)	Mass (g)
Heart rate	25.1±1.4	6.367±1.295	25.1±2.0	6.380±1.500
Body temperature	26.4±1.8	7.306±2.166	23.0±2.8	4.520±1.912
*Ṁ*_O_2__ in air	28.5±2.5	9.456±3.337	23.5±2.3	4.447±1.061
*Ṁ*_O_2__ in water	25.2±1.1	6.178±1.094	27.6±2.2	8.428±2.734
*P*_O_2__ in air	27.0±3.3	7.826±3.749	22.7±3.9	4.227±2.174
*P*_O_2__ in water	25.5±3.1	7.183±2.939	24.2±2.6	5.392±2.087

### Data analyses

All statistical analyses were performed in R v4.0.3 (https://www.r-project.org/). Data were examined for normality of the residuals’ distribution using Shapiro–Wilk tests, and for homogeneity of the residuals' variance using Levene tests (R package ‘car’, Fox and Weisberg, 2019). When assumptions of normality and homoscedasticity were violated, non-parametric tests were applied (summary statistics in Table S1).

Habitat temperature differences between *M. tomentosus* and *T. arcuata* were compared using the Mann–Whitney *U*-test. Heart rate (beats min^−1^) was determined at 1-min intervals along the thermal ramp and plotted against crab body temperature. We discarded the first 10 min of recordings to minimize handling effects on heart performance. We considered the resulting thermal response curves reliable because heart rates across the thermal ramp were consistent with those reported for other decapod crustaceans (Ern et al., 2015; Leung et al., 2023; Levinton et al., 2020). The temperature at which the heart function ceased was used as a proxy for upper lethal temperature (LT) (Marshall et al., 2010). The optimum performance temperature (OPT) for the heart rate, defined as the temperature at which heart rate was maximal, was defined by applying locally weighted scatterplot smoothing (LOWESS; span=0.05, three robustifying iterations) to the heart rate thermal performance curves. The Arrhenius breakpoint temperature (ABT), the temperature at which there is a discontinuity in the slope of the Arrhenius plot and heart rate rapidly decreases (Marshall et al., 2011; Somero, 2005; Stillman and Somero, 1996), was calculated for each individual by plotting the natural log of heart rate against the inverse body temperature (Kelvin) and fitting piece-wise regression models (R package ‘segmented’, Muggeo, 2003, 2008; Fig. S1 and S2, Table S2). The LT and OPT were compared between species using Student's *t*-tests, whereas ABT was compared with the Mann–Whitney *U*-test. Heart rates across temperatures were compared using a mixed-model aligned ranks ANOVA (Wobbroco et al., 2011) from the ‘ARTool’ package (https://CRAN.R-project.org/package=ARTool), with species as a fixed factor, temperature as a continuous covariable, individual as a random effect, and heart rate as the response variable.

*Ṁ*_O_2__ was log-transformed and analysed using a linear mixed-effects model in an ANCOVA framework, with species and medium (aerial versus aquatic) as fixed factors, temperature as a continuous covariable, individual as a random intercept, and oxygen consumption as the response variable. Interactions among temperature, species and medium were tested. Simulation-based residual diagnostics (‘DHARMa’; https://CRAN.R-project.org/package=DHARMa) indicated heteroscedasticity (Fig. S3); the model was therefore re-fit in ‘nlme’ (https://CRAN.R-project.org/package=nlme; Pinheiro and Bates, 2000) with species-specific residual variances via ‘varIdent’. To characterize the temperature–*Ṁ*_O_2__ functional form, we fitted linear, quadratic, exponential and power ordinary least squares (OLS) regressions and selected the best-fitting model using Akaike's information criterion corrected for small samples (AICc; Akaike, 1992; Hurvich and Tsai, 1989; Table S3). Selected models that did not meet the assumptions of homoscedasticity (Breusch–Pagan test; Breusch and Pagan, 1979) were refitted by robust MM-estimator (RLM) regression (‘robustbase::lmrob’; https://CRAN.R-project.org/package=robustbase). Model predictive performance was assessed by 10-fold cross-validation (CV) with 50 repeats in ‘caret’ (Maechler et al., 2024), reporting mean±s.d. of root mean squared error (RMSE), mean absolute error (MAE) and correlation-based *R*^2^ computed on post-resample on held-out test sets from CV. Robust models were selected by minimizing RMSE, MAE and *R*^2^ from psi-function (Huber, Hampel, bisquare) tuned by CV, with Hampel returning the best tuning. Practical significance was evaluated using a pre-specified standardized smallest effect size of interest (SESOI) of *d*=0.5, representing a cautious minimum effect threshold (Cohen, 1988, 1992), applied on the model scale. For each parameter, we computed a standardized effect as the absolute predicted change in the outcome (*Ṁ*_O_2__, *P*_O_2__) over Δ*T*=1°C (for temperature slopes, unit contrasts for categorical terms) by the model residual standard deviation of the fitted model. For RLM fits, residual scale from the model summary was used, and for OLS fits, the residual standard deviation was used. Effects were classified as practically important if their 95% confidence interval for the standardized effect lay entirely beyond *d*=0.5, practically negligible if entirely within ±0.5, and otherwise inconclusive. Outlier influence in significant OLS models was assessed via Cook's distance, leverage and studentized residuals; RLM estimates were compared with OLS to evaluate sensitivity of fixed-effect inferences.

*P*_O_2__ in arterial (*P*a_O_2__) and venous (*P*v_O_2__) haemolymph, as well as the difference (Δ*P*_O_2__=*P*a_O_2__–*P*v_O_2__), were analysed separately. Data were transformed to improve normality (*P*a_O_2__: square root; *P*v_O_2__: natural logarithm; Δ*P*_O_2__: natural logarithm plus 1.2 to remove negative values) and analysed by ANCOVA with species and medium as fixed factors and temperature as a continuous variable. Interactions among covariates and fixed factors were tested. Regression analyses investigating the relationship between temperature and haemolymph *P*_O_2__ were conducted using the same model selection approach as for *Ṁ*_O_2_ _(Table S4).

## RESULTS

### Habitat temperatures

Sediment surface temperature was significantly higher in the habitat of *M. tomentosus* compared with that of *T. arcuata* (Mann–Whitney, *U*=2,343,230, *P*<0.001; Fig. 1). For *T. arcuata*, the mean±s.d., absolute maximum and absolute minimum recorded temperatures were 28.0±1.5°C, 35.5°C and 25.5°C, respectively. In contrast, *M. tomentosus* experienced a mean temperature of 29.8±1.6°C, with a maximum of 37.2°C and a minimum of 27.1°C.

### Cardiac function and thermal limits

**Fig. 2.**
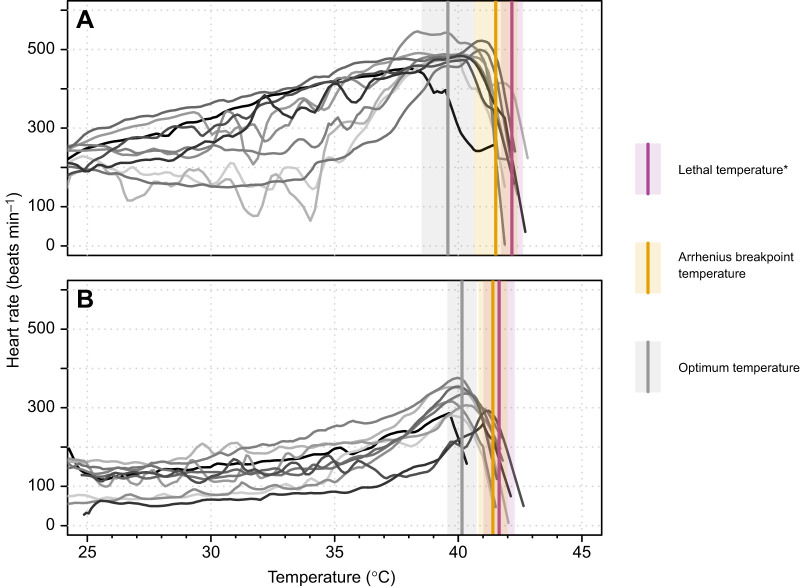
**Thermal performance curves for the heart rates (beats min^−1^, Lowess smoothed) of the study species.** (A) *Tubuca arcuata* and (B) *Macrophthalmus tomentosus*. Each line with a different shade of grey represents a different individual. Purple, yellow and grey vertical lines represent heart performance parameters upper lethal temperature (LT), Arrhenius breakpoint temperature (ABT) and optimum performance temperature (OPT), respectively. Shades indicate standard deviation for heart performance parameters. Asterisk indicates significant difference between species (Student’s *t*-test, *P*<0.05).

Heart rates increased with temperature in both species until a sharp decline was observed after the ABT (Fig. 2). Across all temperatures, *T. arcuata* exhibited higher average heart rates than *M. tomentosus* (mixed-model ART ANCOVA, *F*_1,7558_=50.899, *P*<0.001). The LT was significantly higher in *T. arcuata* compared with *M. tomentosus* (Student’s *t*-test, *t*=2.2644, d.f.=15.945, *P*=0.038; Fig. 2). No differences were observed between species for ABT (Mann–Whitney *U* test, *U*=64, *P*=0.288) or OPT (Student’s *t*-test, *t*=−1.5258, d.f.=13.889, *P*=0.149) (Fig. 2).

### Metabolic rates

**Fig. 3.**
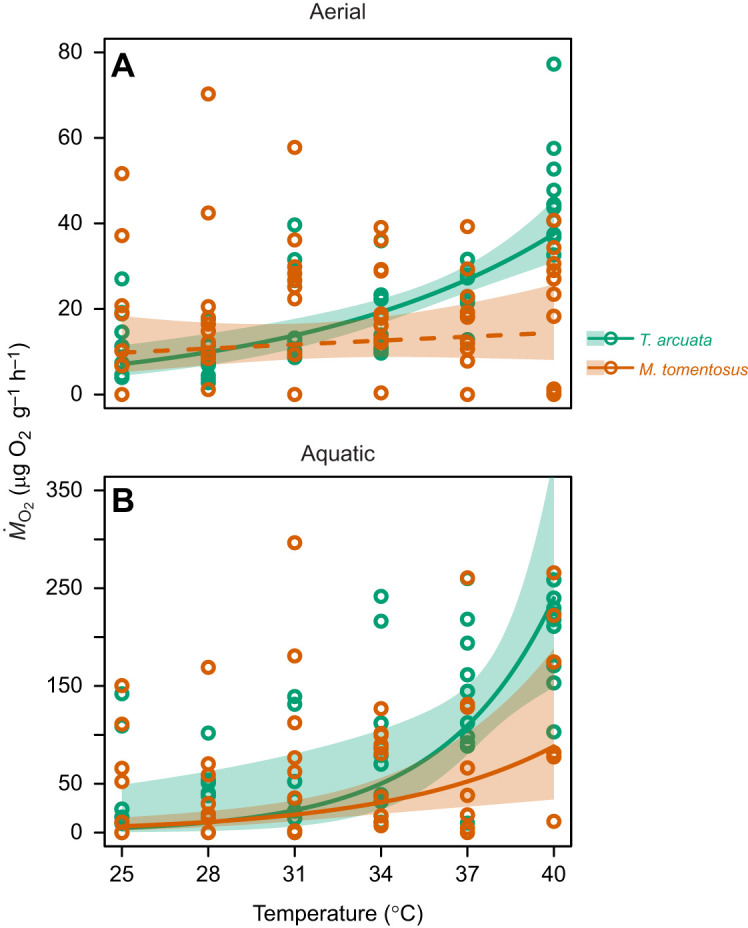
**Relationship between temperature and metabolic rate (*Ṁ*_O_2__) in *Tubuca arcuata* and *Macrophthalmus tomentosus* in air and water.** (A) Air and (B) water. Continuous and dashed lines represent significant and non-significant regressions, respectively, with their 95% confidence intervals (see Table 2). Colour-coded points represent individual raw *Ṁ*_O_2__ values at each temperature.

*Ṁ*_O_2__ increased exponentially with temperature in *T. arcuata* in air and water and in *M. tomentosus* in water (Table 2, Fig. 3), with 95% confidence intervals for the temperature effect excluding zero and exceeding the prespecified SESOI, indicating statistical and practical significance. CV performance supported these patterns (*T. arcuata* air: RMSE=0.590±0.226, MAE=0.451±0.175, correlation-based *R*^2^=0.627; *T. arcuata* water: RMSE=1.498±0.344, MAE=1.185±0.328, *R*^2^=0.151; *M. tomentosus* water: RMSE=1.930±0.347, MAE=1.697±0.331, *R*^2^=0.328). In the OLS fit for *M. tomentosus* in water, influential points were identified; a robust MM-estimator fit yielded similar conclusions (intercept β_0_=−3.060±1.847, temperature slope β_1_=0.195±0.053, robust Wald χ^2^_1_= 13.26, adj. *R*^2=^0.194, *P*<0.001). For both species, *Ṁ*_O_2__ was significantly higher in water than in air at elevated temperatures, particularly above 34°C (two-way mixed model ANCOVA, *F*_1,196_=19.003, *P*<0.001; Fig. 3). A significant interaction was found between temperature and species, with *T. arcuata Ṁ*_O_2__ being more responsive to temperature increases (two-way mixed-model ANCOVA, *F*_1,196_=7.518, *P*=0.007; Fig. 3).

**Table 2.** Summary of the ordinary least squares (OLS) and robust MM-estimator (RLM) regression analysis for the relationship between temperature and oxygen consumption in both aerial and aquatic media

Species	Medium	Method	Form	β_0_	β_1_	Equation	Statistic (*F*/Wald χ^2^)	Adj. *R*^2^	*P*
*Tubuca arcuata*	Air	RLM	Exponential	−0.813±0.752	0.111±0.021	0.444*e*^0.111*x*^	28.803	0.558	**<0.001**
	Water	RLM	Exponential	−4.951±3.398	0.261±0.090	0.007*e*^0.261*x*^	8.443	0.487	**=0.003**
*Macrophthalmus tomentosus*	Air	OLS	Power	−0.374±3.678	0.825±1.059	0.688*x*^0.825^–1	10.847	−0.007	0.439
	Water	OLS	Exponential	−2.491±1.610	0.174±0.049	0.083*e*^1.174*x*^	12.68	0.165	**<0.001**

Statistically significant regressions (*P*<0.05) are shown in bold.

### Partial pressure of oxygen in the haemolymph

**Fig. 4.**
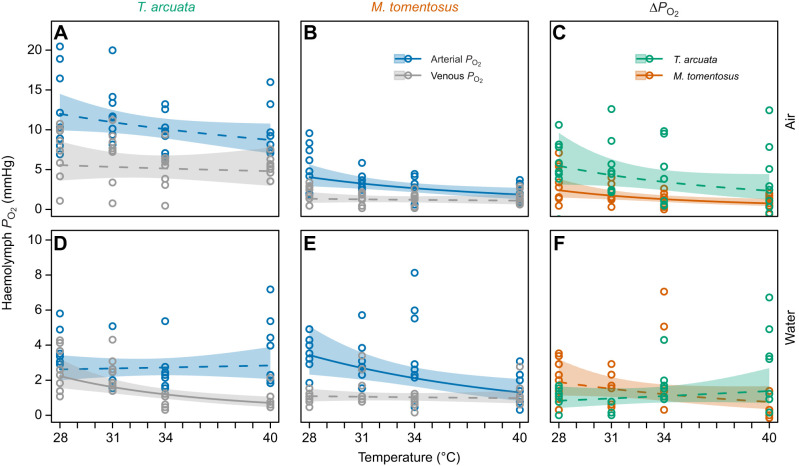
**Relationships between temperature and arterial blood partial pressure of oxygen (*P*a_O_2__), venous *P*_O_2__ (*P*v_O_2__) and their difference (Δ*P*_O_2__) in the aerial and aquatic media.** (A–C) Air and (D–F) water. Continuous and dashed lines represent significant and non-significant regressions, respectively, with their 95% confidence intervals (see Table 3). Colour-coded points represent individual raw *P*_O_2__ values at each temperature.

The relationship between *P*a_O_2__ and temperature was best described by a power regression in air and by an exponential decrease in water for both species (Table 3, Fig. 4). These relationships were only significant for *M. tomentosus* in air (the 95% CI lay entirely beyond the SESOI and excluded zero, indicating statistical and practical significance; CV performance: RMSE=0.552±0.197, MAE=0.481±0.184, *R*^2^=0.569; Table 3, Fig. 4B). In water, the temperature effect on *M. tomentosus P*a_O_2_ _was statistically significant but small; because the entire 95% CI [−0.138, −0.024] lay within the SESOI bounds, practical equivalence to a negligible effect is supported under this threshold (CV performance: RMSE=0.648±0.308, MAE=0.524±0.270, *R*^2^=0.591; Table 3, Fig. 4D). Increasing temperature significantly reduced *P*a_O_2_ _(two-way ANCOVA, *F*_1,120_=13.909, *P*<0.001), except in *T. arcuata* when in water (Fig. 4A,B,D,E). Both species and medium had significant effects on *aP*_O_2__ (*F*_1,120_=83.416, *P*<0.001 and *F*_1,120_=100.100, *P*<0.001, respectively), as did their interaction (*F*_1,120_=65.359, *P*<0.001). Notably, *T. arcuata* exhibited higher *P*a_O_2__ in air compared with water, whereas *M. tomentosus* showed consistently low *P*a_O_2__ in both air and water (Fig. 4A,B,D,E).

**Table 3.** Summary of the best-fit OLS regression analysis for the relationship between temperature and arterial and venous blood partial pressure of oxygen (*P*_O_2__) and the difference between them (Δ) in both air and water

Species	Medium	Blood *P*_O_2__	Form	β_0_	β_1_	Equation	*F*	Adj. *R*^2^	*P*
*Tubuca arcuata*	Air	Arterial	Power	5.468±1.549	−0.895±0.443	237.081*x*^−0.895^	4.085	0.090	0.052
		Venous	Power	3.095±3.513	−0.414±1.004	22.087*x*^−0.414^	0.169	−0.027	0.683
		Δ	Power	9.634±4.660	−2.381±1.333	9.634*x*^−2.381^	3.192	0.070	0.085
	Water	Arterial	Exponential	0.769±0.644	0.007±0.019	2.158*e*^0.007*x*^	0.128	−0.029	0.723
		Venous	Power	11.625±2.983	−3.247±0.853	1.12×10^5^*x*^−3.247^	14.490	0.303	**<0.001**
		Δ	Exponential	−1.378±1.486	0.043±0.043	−0.252*e*^−0.043*x*^	0.9693	−0.001	0.334
*Macrophthalmus tomentosus*	Air	Arterial	Power	8.649±2.709	−2.175±0.775	5704.439*x*^−2.175^	7.888	0.182	**0.009**
		Venous	Power	2.084±3.992	−0.538±1.141	8.046*x*^−0.538^	0.222	−0.027	0.641
		Δ	Power	11.652±3.941	−3.233±1.129	1.16×10^5^*x*^−3.233^	8.194	0.204	**0.008**
	Water	Arterial	Exponential	3.498±0.934	−0.081±0.028	33.060*e*^−0.808*x*^	8.418	0.193	**0.007**
		Venous	Power	1.149±2.650	−0.319±0.758	3.158*x*^−0.319^	0.177	−0.027	0.677
		Δ	Exponential	2.765±1.421	−0.076±0.044	15.88*e*^−0.076*x*^	30.21	0.070	0.094

Statistically significant regressions (*P*<0.05) are shown in bold.

*P*v_O_2__ also followed power regressions with temperature, but were significant only for *T. arcuata* in water (95% CI for slope entirely beyond the SESOI, indicating statistical and practical significance; CV performance: RMSE=0.613±0.209, MAE=0.526±0.203, *R*^2^=0.656; Table 3). Influential observations were identified via Cook's distance, and MM-estimator robust regression yielded similar significance (β_0_=12.007±2.918, β_1_=−3.354±0.842, adj. *R*^2=^0.303, *P*<0.001). Temperature significantly affected *P*v_O_2__ (two-way ANCOVA, *F*_1,120_=4.931, *P*<0.028), with *P*v_O_2__ decreasing at higher temperatures in both species and media (Fig. 4A,B,D,E). The main effects of species and medium were significant (*F*_1,120_=45.465, *P*<0.001 and *F*_1,120_=39.937, *P*<0.001, respectively), as was the interaction (*F*_1,120_=23.121, *P*<0.001). *Tubuca arcuata* had higher *P*v_O_2__ in air than in water, whereas *M. tomentosus* displayed low *P*v_O_2__ across both media, particularly at high temperatures. Additionally, *T. arcuata* exhibited higher *P*v_O_2_ _than *M. tomentosus* in both air and water (Fig. 4A,B,D,E).

For Δ*P*_O_2__, in both species the relationship with temperature followed a power regression in air and an exponential regression, with statistical significance observed for *M. tomentosus* in air only; the 95% CI lay entirely beyond the SESOI in magnitude, establishing practical significance (CV performance: RMSE=0.792±0.291, MAE=0.701, *R*^2^=0.621; Table 3). Δ*P*_O_2__ was significantly higher in air than in water for both species (two-way ANCOVA, *F*_1,120_=7.067, *P*=0.030). This was more pronounced in *T. arcuata* (Fig. 4C). Neither temperature nor species had a significant effect on Δ*P*_O_2__ (*F*_1,120_=0.192, *P*=0.662 and *F*_1,120_=0.005, *P*=0.943, respectively), but the interaction between factors was significant (*F*_1,120_=5.546, *P*=0.020). In *T. arcuata*, Δ*P*_O_2__ decreased with increasing temperature in air and remained relatively constant in water (Fig. 4C,F), whereas in *M. tomentosus*, Δ*P*_O_2__ decreased with increasing temperature in both media (Fig. 4B,C,E,F).

## DISCUSSION

Thermal constraints on aerobic metabolism and energy budgets can reduce performance and fitness, thereby exerting evolutionary pressure that favours the evolution of air-breathing adaptations in tropical climates (Giomi et al., 2014). We investigated how air-breathing influences thermal tolerance and physiological vulnerability in two closely related intertidal crabs with contrasting respiratory modes. By directly comparing cardiac, metabolic and haemolymph oxygenation responses under aerial and aquatic exposure, we build on the known benefits of air-breathing to provide a more mechanistic understanding of its adaptive significance across distinct thermal habitats. Our results support the view that air-breathing enhances thermal resilience in ocypodid crabs and highlight respiratory mode as a key trait shaping habitat partitioning and microhabitat exploitation in the intertidal zone (Fusi et al., 2015; Jimenez et al., 2025).

Both *T. arcuata* and *M. tomentosus* exhibited maximal heart rates and metabolic demands at high temperatures (>40°C), reflecting a stress response to acute heating (de Pirro et al., 1999; Maus et al., 2021; McGaw and Reiber, 2015; Rovero et al., 2000). Elevated heart rate can increase cardiac output and convective haemolymph flow to sustain tissue oxygen delivery during warming and by increasing perfusion of exposed body surfaces (e.g. gills, branchiostegites, appendages), facilitate heat exchange and oxygen uptake with the surrounding medium (De Wachter and McMahon, 1996; Goudkamp et al., 2004).

The metabolic responses to temperature further revealed distinct respiratory capabilities between species. *Tubuca arcuata* demonstrated exponential increases in *Ṁ*_O_2__ with temperature in both air and water, indicating maintained respiratory function across media and supporting its classification as a functional air-breather (Flemister and Flemister, 1951; Hawkins et al., 1982; Jimenez and Bennett, 2005). In contrast, *M. tomentosus* showed increased *Ṁ*_O_2__ only when submerged, indicating a limited ability to modulate oxygen uptake in air despite thermal sensitivity in aquatic conditions. Notably, nine out of ten *M. tomentosus* individuals exhibited impaired motor responses after aerial *Ṁ*_O_2__ trials, whereas all individuals recovered normally after aquatic trials, highlighting the importance of water for respiratory function in this species. By contrast, *T. arcuata* maintained motor responses post-experimentation in both media. Both species exhibited higher *Ṁ*_O_2__ in water than in air, consistent with patterns observed in other ocypodids (Flemister and Flemister, 1951; Teal, 1959; Veerannan, 1974) and macrophthalmid species (Hawkins et al., 1982), although this pattern is not universal among intertidal crabs and can vary even at the level of family, including in the Ocypodidae (Eshky et al., 1988; Fusi et al., 2016; Innes et al., 1986; Jimenez and Bennett, 2005; Teal, 1959).

Our haemolymph oxygenation analyses reveal fundamental differences in respiratory capacity between the two species during aerial exposure. *Tubuca arcuata* maintained significantly higher *P*a_O_2__ and *P*v_O_2__ than *M. tomentosus* when breathing air, with both measures showing minimal temperature sensitivity across our experimental range (28–40°C). This physiological stability indicates that *T. arcuata* can sustain adequate oxygen delivery to tissues even at temperatures approaching its thermal limits, reflecting efficient air-breathing capabilities. In contrast, *M. tomentosus* exhibited a decline in *P*a_O_2__ with increasing temperature during aerial exposure, coupled with consistently low *P*v_O_2__, indicating limited oxygen uptake from air and progressive tissue hypoxia. These patterns align with the established framework that *P*a_O_2__ reflects gas exchange efficiency whereas *P*v_O_2__ indicates the balance between oxygen supply and tissue demand (Fusi et al., 2016; Giomi and Pörtner, 2013; Giomi et al., 2014). The maintained oxygenation in *T. arcuata* during aerial heating supports its capacity to remain active in warm, upper-shore environments. Conversely, the declining oxygen availability in *M. tomentosus* underscores its fundamental dependence on water for respiration and highlights its vulnerability to hypoxemia and metabolic limitation when emersed at high temperatures (Pörtner, 2001; Pörtner and Knust, 2007). The depleted *P*_O_2__ likely constrains aerobic scope for activity and reproductive output at sublethal but elevated temperatures (Pörtner et al., 2017; Rubalcaba et al., 2020). Thus, blood *P*_O_2__ may be a promising proxy for assessing thermal vulnerability to warming, warranting validation in natural populations under realistic thermal regimens.

Lower oxygen solubility in water, and the metabolic costs of maintaining homeostasis during aquatic respiration imposes physiological constraints on both air- and water-breathing crabs at elevated temperatures (Dejours, 1981; O'Mahoney and Full, 1984). When submerged, *T. arcuata* exhibited lower *P*a_O_2__ compared with aerial conditions, yet *P*a_O_2_ _remained stable with increasing temperature, indicating efficient oxygenation, similar to the congeneric *T. urvillei* (Fusi et al., 2015). In contrast, *M. tomentosus* showed reduced blood oxygenation in water at high temperatures, implying a limited capacity to oxygenate its arterial blood compared with fiddler crabs. In both species, *P*v_O_2__ declined with increasing water temperature, indicating haemolymph oxygen depletion at high temperatures. These patterns, consistent with previous results on submerged intertidal brachyurans, highlight the adaptive advantage of breathing air under thermal stress (Fusi et al., 2015, 2016; Giomi et al., 2014).

Our results indicate that *T. arcuata* sustains metabolic function and maintains higher haemolymph *P*_O_2__ at elevated temperatures during emersion, enabling aerobic activity and resilience in the upper intertidal zone, where water is scarce during low tide. This physiological advantage allows fiddler crabs to maintain fitness under prolonged aerial exposure and desiccation stress by sustaining their oxygen supply. Behavioural adaptations such as burrow retreat and evaporative cooling from wetted body surfaces further enhance thermal tolerance during emersion (Levinton et al., 2015; Thurman, 1998). Additionally, traits such as capillary wicking and dynamic carapace colour change help regulate body temperature and water balance, complementing the improved blood oxygenation we observed (Silbiger and Munguia, 2008; Thompson et al., 1989; Wolcott, 1984). *Tubuca arcuata*'s superior thermal tolerance and adaptation to drier conditions allows the species to colonize and thrive in the upper intertidal and mangrove zones, where desiccation stress is high and water access is periodic. In contrast, *M. tomentosus* exhibits progressive oxygenation constraints at elevated temperatures, limiting its ability to sustain activity during thermal stress. Critically, *M. tomentosus* remains water-dependent and cannot exploit drier microhabitats where *T. arcuata* thrives. These findings establish that respiratory physiology is a primary driver of thermal tolerance and niche partitioning in these species, whereas other factors such as microhabitat structure, substrate type and resource availability operate within these physiological boundaries. Ultimately, *T. arcuata*'s physiological capacity provides a thermal safety margin that may prove critical under future warming scenarios, given the species is not constrained by mortality during different ontogenetic stages (Marochi et al., 2021; Vorsatz et al., 2022).

Contrastingly, the reduced haemolymph oxygen at high temperatures in *M. tomentosus* indicates higher vulnerability to warming, as rising temperatures increase metabolic oxygen demand while simultaneously decreasing oxygen solubility in water (Rubalcaba et al., 2020), creating a dual physiological squeeze that will only intensify under future warming. Critically, when we contextualize haemolymph *P*_O_2__ against the temperatures each species naturally experiences, we find that *M. tomentosus* already exhibits oxygen deficits in its natural habitat. To exploit its microhabitat, this species already relies on behavioural thermoregulation, such as seeking refuge in burrows or retreating into water, which constrains its time budget. However, under current and projected warming, this safety margin will be compressed, intensifying reliance on refugia and potentially exhausting available ones. Our findings on blood oxygen depletion and thermal limits reveal that *M. tomentosus* populations currently operate near the physiological threshold where oxygen delivery fails, meaning that thermoregulatory behaviour is essential for the crabs' survival, but ultimately leaving little margin for thermal increase.

In summary, our comparative analysis reveals that air-breathing physiology represents a fundamental mechanism enabling differential thermal tolerance and niche partitioning through operational boundaries that constrain habitat occupancy and thermal limits in intertidal crabs. We demonstrate that direct aerial respiration allows fiddler crabs to maintain metabolic performance and sustain haemolymph oxygenation at temperatures that incapacitate their water-breathing relatives. These findings furnish experimental support for the OCLTT framework in crustaceans, identifying respiratory mode as a critical life-history trait determining species resilience under warming climates (Jimenez et al., 2022, 2025; Leiva et al., 2019; Payne and Smith, 2017). By integrating physiological assays with habitat temperature profiles, this study advances our mechanistic understanding of differential thermal tolerance and resilience in intertidal species. Our framework revealed asymmetric climate vulnerability. Water-breathing species, such as *M. tomentosus*, which currently operate near their physiological oxygen-delivery thresholds, face an elevated risk of local extinction under warming scenarios. In contrast, air-breathing species such as *T. arcuata*, which possess wider physiological safety margins, are likely to expand their distribution or maintain populations as thermal refugia become scarcer. Recent fiddler crab range expansions in response to warming support this prediction (Arakaki et al., 2020; Jimenez et al., 2024; Johnson, 2014). Differential responses suggest that future intertidal communities may be substantially restructured, with water-breathing crabs potentially declining in warm-exposed zones. Our work thus provides a mechanistic basis for predicting climate vulnerability and prioritizing conservation efforts towards threatened water-breathing taxa before local extinctions eliminate key ecosystem functions.

## Supplementary Material

10.1242/jexbio.251854_sup1 Supplementary information
