# Supplementary material for: Divergent respiratory modes drive differences in heat tolerance and habitat use among tropical intertidal crabs
Source: J Exp Biol. 2026 Apr 22;229(8):jeb251854. doi: 10.1242/jeb.251854 (PMC13143215; doi:10.1242/jeb.251854)
Supplement: Supplementary information [file jexbio-229-251854-s1.pdf]

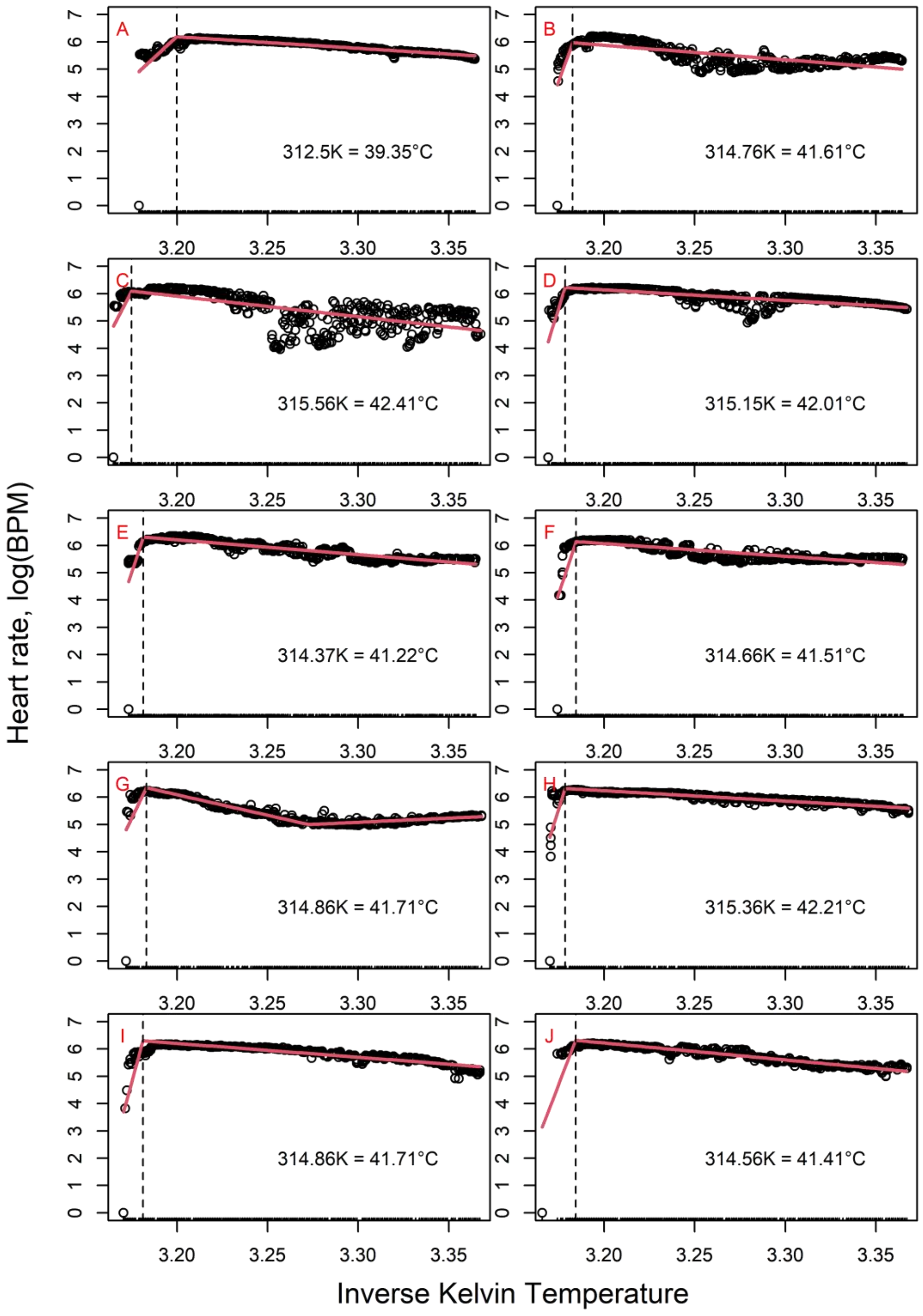

Fig. S1. Broken stick regressions of heart rate (beats min^{-1}) in *Tubuca arcuata* vs. inverse temperature (K^{-1}). Red lines show piecewise regression fits; black circles represent raw data points; and breakpoint temperature is shown in both Kelvin and Celsius.

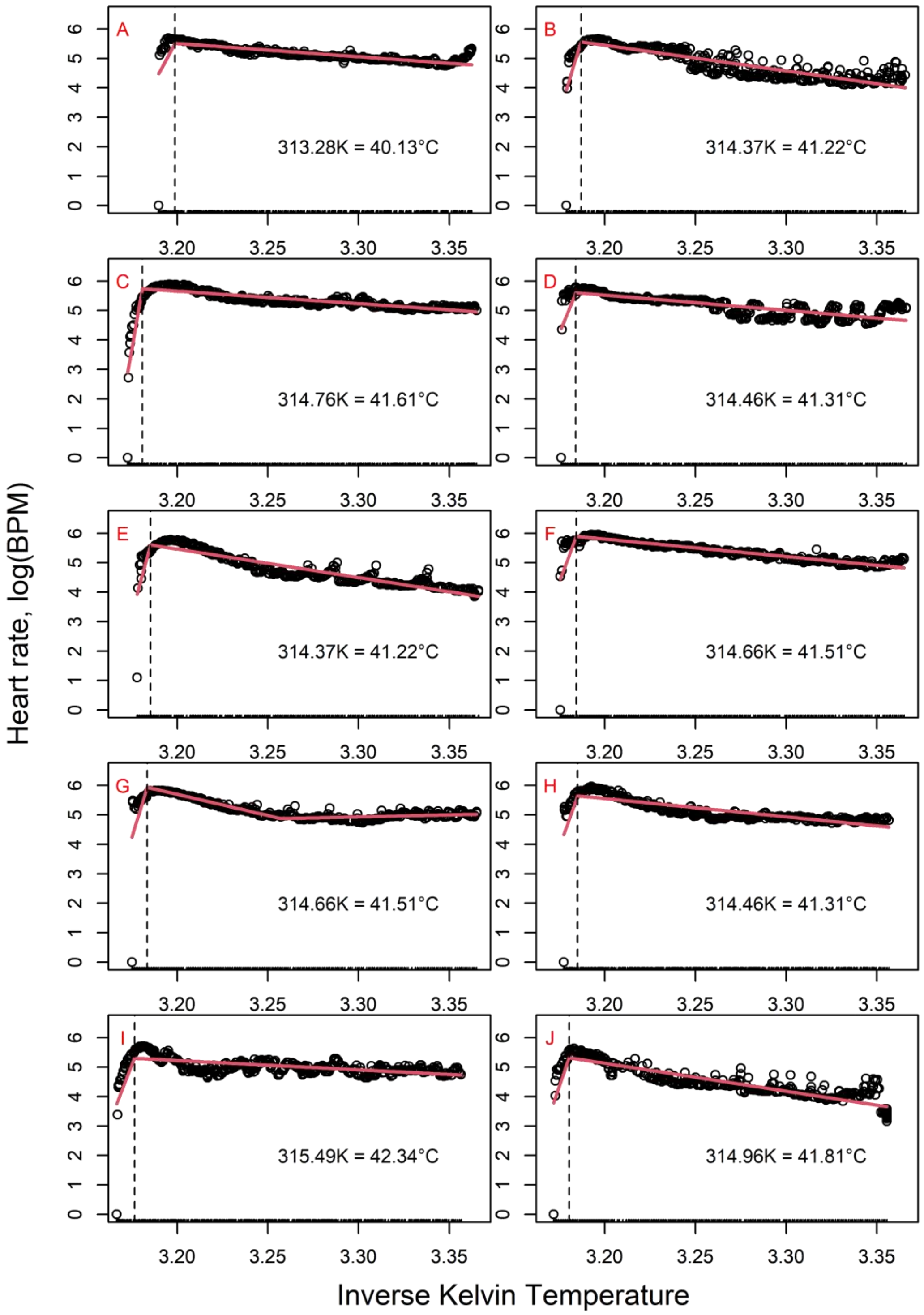

Fig. S2. Broken stick regressions of heart rate (beats min^{-1}) in *Macrophthalmus tomentosus* vs. inverse temperature (K^{-1}). Red lines show piecewise regression fits; black circles represent raw data points; and breakpoint temperature is shown in both Kelvin and Celsius.

Journal of Experimental Biology • Supplementary information

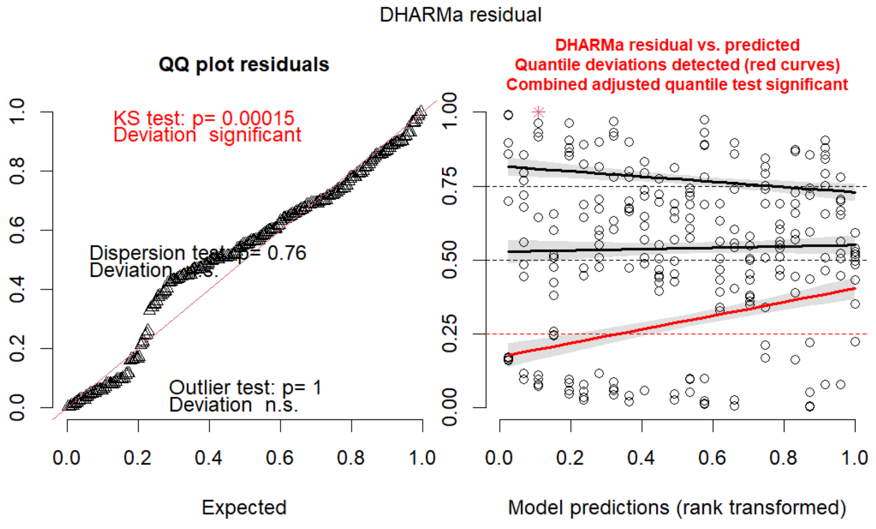

Fig. S3. Diagnostic plots for respiration (MO_2) ANCOVA model. Left: DHARMa Q-Q plot assessing normality. Right: Residuals vs. fitted values revealing heteroscedasticity.

Table S1. Summary of statistical tests for normality (Shapiro–Wilk W; Anderson–Darling A for high *n*), homoscedasticity (Levene's test), and optimal data transformations used in primary regression analyses.

Variable model	Data transformation	Homoscedasticity test			Normality check	
		DF	F	P	Test (A/W)	P
Habitat temperature	Original	1/7236	13.278	<0.001	A = 164.35	<0.001
	Log	1/7236	48.747	<0.001	A = 128.6	<0.001
LT	Original	1/18	0.251	0.622	W = 0.944	0.286
	Log	1/18	0.279	0.604	W = 0.280	0.240
ABT	Original	1/18	0.289	0.598	W = 0.002	0.002
	Log	1/18	0.286	0.599	W = 0.816	0.001
OPT	Original	1/18	2.929	0.104	W = 0.971	0.784
	Log	1/18	3.063	0.097	W = 0.972	0.971
Heart rates	Original	9/90	1.544	0.145	W = 0.923	<0.001
	Log	9/90	2.240	0.026	W = 0.843	<0.001
aPO2	Original	15/112	3.286	<0.001	W = 0.939	<0.001
	Sqrt	15/112	1.2302	0.260	W = 0.985	0.175
vPO2	Original	15/112	3.805	<0.001	W = 0.898	<0.001
	Log	15/112	0.660	0.818	W = 0.910	<0.001
ΔPO2	Original	15/112	3.050	<0.001	W = 0.930	<0.001
	Log	15/112	0.834	0.639	W = 0.605	<0.001

Journal of Experimental Biology • Supplementary information

Table S2. Model parameters for broken-stick regressions (slopes before and after breakpoint) showing estimates, standard errors (SE), t-values, 95% confidence intervals (CI), and Adjusted R^2. Individual (Ind) letters correspond to those in Fig. S1 for *T. arcuata*, and Fig. S2 for *M. tomentosus*.

	Ind	Coefficient	Estimate	SE	*t* value	CI lower	CI upper	Adj. R^2
Macrophthalmus tomentosus	A	Slope 1	115.400	26.103	4.421	64.056	166.750	0.375
		Slope 2	-4.481	0.334	-13.394	-5.139	-3.8228	
	B	Slope 1	201.880	25.269	7.989	152.190	251.570	0.671
		Slope 2	-8.703	0.331	-26.282	-9.354	-8.052	
	C	Slope 1	352.270	17.475	20.158	317.900	386.630	0.757
		Slope 2	-4.237	0.208	-20.363	-4.646	-3.828	
	D	Slope 1	151.380	28.038	5.3992	96.249	206.510	0.427
		Slope 2	-5.199	0.327	-15.884	-5.842	-4.555	
	E	Slope 1	226.780	26.704	8.492	174.260	279.290	0.801
		Slope 2	-9.661	0.257	-37.543	-10.167	-9.155	
	F	Slope 1	167.740	25.520	6.495	115.560	215.930	0.585
		Slope 2	-5.923	0.273	-21.702	-6.460	-5.387	
	G	Slope 1	203.760	23.664	8.611	157.220	250.300	0.590
		Slope 2	-14.376	1.038	-13.849	-16.417	-12.335	
	H	Slope 1	173.500	26.731	6.490	120.920	226.070	0.522
		Slope 2	-6.181	0.333	-18.555	-6.836	-5.526	
	I	Slope 1	157.500	24.373	6.462	109.580	205.430	0.383
		Slope 2	-3.155	0.293	-10.778	-3.731	-2.580	
	J	Slope 1	179.110	26.833	6.675	126.340	231.880	0.708
		Slope 2	-9.488	0.330	-28.777	-10.137	-8.840	
Tubuca arcuata	A	Slope 1	61.212	6.967	8.786	47.513	74.911	0.450
		Slope 2	-4.242	0.312	-13.603	-4.855	-3.6284	
	B	Slope 1	183.560	28.130	6.525	128.250	238.870	0.410
		Slope 2	-5.318	0.367	-14.487	-6.040	-4.5963	
	C	Slope 1	128.280	41.057	3.124	47.563	208.990	0.372
		Slope 2	-7.491	0.490	-15.291	-8.454	-6.528	
	D	Slope 1	-213.550	21.761	9.814	170.770	256.340	0.483
		Slope 2	-3.869	0.276	-14.027	-4.411	-3.326	
	E	Slope 1	201.090	21.872	9.194	158.090	244.090	0.579
		Slope 2	-5.403	0.265	-20.391	-5.924	-4.882	
	F	Slope 1	199.050	20.344	9.784	159.050	239.040	0.514
		Slope 2	-4.6279	0.2821	-16.405	-5.182	-4.073	
	G	Slope 1	136.700	19.528	7.000	98.310	175.100	0.668
		Slope 2	-15.031	0.779	-19.292	-16.563	-13.499	
	H	Slope 1	211.900	22.487	9.423	167.690	256.120	0.497
		Slope 2	-3.793	0.268	-14.137	-4.321	-3.266	
	I	Slope 1	244.910	18.348	13.348	208.840	280.990	0.658
		Slope 2	-5.073	0.240	-21.113	-5.546	-4.601	
	J	Slope 1	172.660	13.397	12.889	146.320	199.010	0.723
		Slope 2	-6.214	0.226	-27.441	-6.659	-5.769	

Table S3. Best model selection for MO_2 based on Akaike Information Criterion corrected for small sample sizes (AICc). K = number of estimated parameters.

Species	Medium	Model	K	AICc	ΔAICc	AICc weight
Tubuca arcuata	Air	Exponential	2	114.747	0	0.662
		Power	2	116.094	1.347	0.338
		Quadratic	3	446.995	332.248	<0.001
		Linear	2	462.061	347.314	<0.001
	Water	Exponential	2	224.655	0	0.556
		Power	2	225.108	0.454	0.443
		Quadratic	3	663.405	438.750	<0.001
		Linear	2	666.470	441.815	<0.001
Macrophthalmus tomentosus	Air	Power	2	207.161	0	0.519
		Exponential	2	207.216	0.155	0.481
		Linear	2	505.951	298.790	<0.001
		Quadratic	3	507.133	299.971	<0.001
	Water	Exponential	2	253.910	0	0.549
		Power	2	254.307	0.397	0.451
		Quadratic	3	736.297	482.387	<0.001
		Linear	2	742.285	488.375	<0.001

Journal of Experimental Biology • Supplementary information

Table S4. Best arterial, venous, and delta (arterial – venous) PO_2 model selection by Akaike Information Criterion corrected for small-sample (AICc). K = number of parameters.

Species	Medium	Blood PO_2	Model	K	AICc	ΔAICc	AICc weight
Tubuca arcuata	Air	Arterial	Power	2	23.943	0	0.512
			Exponential	2	24.037	0.094	0.488
			Linear	2	180.774	156.831	<0.001
			Quadratic	3	183.262	159.319	<0.001
		Venous	Power	2	76.340	0	0.505
			Exponential	2	76.380	0.041	0.495
			Linear	2	159.817	83.478	<0.001
			Quadratic	3	162.171	85.831	<0.001
		Δ	Power	2	85.079	0	0.523
			Exponential	2	85.267	0.188	0.476
			Linear	2	179.097	94.018	<0.001
			Quadratic	3	181.516	96.437	<0.001
	Water	Arterial	Exponential	2	48.411	0	0.509
			Power	2	48.484	0.073	0.491
			Quadratic	3	119.872	71.461	<0.001
			Linear	2	122.236	73.825	<0.001
		Venous	Power	2	65.860	0	0.609
			Exponential	2	66.748	0.889	0.391
			Quadratic	3	93.272	27.411	<0.001
			Linear	2	95.646	29.786	<0.001
		Δ	Exponential	2	81.937	0	0.503
			Power	2	81.964	0.027	0.497
			Linear	2	119.150	37.213	<0.001
			Quadratic	3	121.022	39.085	<0.001
Macrophthalmus tomentosus	Air	Arterial	Power	2	59.703	0	0.568
			Exponential	2	60.254	0.552	0.431
			Quadratic	3	132.944	73.242	<0.001
			Linear	2	135.962	76.259	<0.001
		Venous	Power	2	84.503	0	0.390
			Exponential	2	84.596	0.092	0.373
			Quadratic	3	86.236	1.732	0.164
			Linear	2	87.855	3.351	0.073
		Δ	Power	2	74.151	0	0.512
			Exponential	2	74.251	0.999	0.487
			Quadratic	3	121.612	47.461	<0.001
			Linear	2	121.983	47.830	<0.001
	Water	Arterial	Exponential	2	72.264	0	0.568
			Power	2	72.811	0.548	0.432
			Quadratic	3	128.156	55.892	<0.001
			Linear	2	129.125	56.861	<0.001
		Venous	Power	2	58.287	0	0.502
			Exponential	2	58.309	0.021	0.496
			Linear	2	69.877	11.589	0.001
			Quadratic	3	72.313	14.026	<0.001
		Δ	Exponential	2	77.727	0	0.540
			Power	2	78.049	0.322	0.460
			Quadratic	3	122.386	44.659	<0.001
			Linear	2	124.129	46.402	<0.001
